# The Experimental Investigation on Mechanics and Damage Characteristics of the Aeolian Sand Paste-like Backfill Materials Based on Acoustic Emission

**DOI:** 10.3390/ma15207235

**Published:** 2022-10-17

**Authors:** Xiaoping Shao, Chuang Tian, Chao Li, Zhiyu Fang, Bingchao Zhao, Baowa Xu, Jianbo Ning, Longqing Li, Renlong Tang

**Affiliations:** 1 Energy School, Xi’an University of Science and Technology, Xi’an 710054, China; 2 Key Laboratory of Western Mines and Hazards Prevention, Ministry of Education of China, Xi’an 710054, China; 3 Shaanxi Coal and Chemical Technology Institute Co., Ltd., Xi’an 710054, China

**Keywords:** backfill mining, loading rate, mechanical properties, acoustic emission, cumulative ringing count, damage constitutive model

## Abstract

With the wide application of the filling mining method, it is necessary to consider the influence of rock activity on the filling body, reflected in the laboratory, that is, the influence of loading rate. Therefore, to explore the response characteristics of loading rate on the mechanical and damage characteristics of aeolian sand paste filling body, DNS100 electronic universal testing machine and DS5-16B acoustic emission (AE) monitoring system were used to monitor the stress–strain changes and AE characteristic parameters changes of aeolian sand paste filling body during uniaxial compression, and the theoretical model of filling sample damage considering loading rate was established based on AE parameters. The experimental results show that: (1) With the increase in loading rate, the uniaxial compressive strength and elastic modulus of aeolian sand paste-like materials (ASPM) specimens are significantly improved. ASPM specimens have ductile failure characteristics, and the failure mode is unidirectional shear failure → tensile failure → bidirectional shear failure. (2) When the loading rate is low, the AE event points of ASPM specimens are more dispersed, and the large energy points are less. At high loading rates, the AE large energy events are more concentrated in the upper part, and the lower part is more distributed. (3) The proportion of the initial active stage is negatively correlated with the loading rate, and the proportion of the active stage is positively correlated with the loading rate. The total number of AE cumulative ringing decreases with the increase in loading rate. (4) Taking time as an intermediate variable, the coupling relationship between ASPM strain considering loading rate and the AE cumulative ringing count is constructed, and the damage and stress coupling model of ASPM specimen considering loading rate is further deduced. Comparing the theoretical model with the experimental results shows that the model can effectively reflect the damage evolution process of ASPM specimens during loading, especially at high loading rates. The research results have significant reference value for subsequent strength design of filling material, selection of laboratory loading rate and quality monitoring, and early warning of filling body in goaf.

## 1. Introduction

As an environmentally friendly mining method, the filling mining method can improve resource recovery rate, control rock migration and surface subsidence, treat solid waste accumulation, improve stope environment and prolong mine service life so as to reduce the influence and damage of resource mining on natural, social and living environment [1,2,3,4,5,6]. The technology has been successfully applied in various engineering environments in many countries [7,8,9]. The Yushenfu mining area in northern Shaanxi is located at western China’s edge of the Maowusu Desert. The surface of the area is covered with a large amount of aeolian sand, and there are many power plants around the mining area. These power plants will produce a large amount of solid waste in the production process, such as fly ash and slag. The accumulation of the solid waste seriously pollutes the ecological environment, and it is necessary to dispose of the solid waste reasonably. Therefore, scholars have proposed a new filling material for the Yushenfu mining area—aeolian sand paste filling material, in which aeolian sand as aggregate, cement and fly ash as cementitious materials [10]. Currently, many coal mines in the mining area are using this filling material to fill the goaf. The filling mainly transports the filling body with bearing characteristics to the goaf after resource mining to support, let the pressure, and prevent rock deformation, to control large area roof and ground pressure activities [11,12,13]. A filling body is a key to ensuring the stope’s safety and stability. Its strength is the core of mechanical problems of filling the body and is also the focus and hotspot of many scholars [14,15,16,17].

The material’s mechanical properties vary with the loading rate, mainly because the loading rate affects the storage characteristics of the elastic energy of the material itself. Komurlu [18], Fujita [19], Huang [20], Cao [21], Yang [22], and Ma [23] have explored the influence of different loading rates on the mechanical behaviour of rock materials. It is believed that changing the loading rate can influence the stress–strain curve, uniaxial compressive strength, peak strain, and failure mode. Pedersen [24], Vidya [25], Ma [26], Dang [27], Zhang [28], Rezaei [29] et al. conducted tests on concrete materials at different loading rates and clarified that the loading rate also impacted the mechanical properties of concrete materials.

The damage study of cemented backfill is one of the most basic and essential research contents in backfill mechanics. Zhao et al. [30] built uniaxial compression damage constitutive model based on Weibull distribution. Based on the energy dissipation theory and damage mechanics theory, Hou et al. [31] constructed the damage constitutive model of cemented tailings backfill considering the curing age. Tu et al. [32] constructed the damage constitutive model of cemented tailings backfill (CTB) under uniaxial compression based on Weibull distribution, strain equivalent principle, and damage mechanics theory. They verified the model’s validity using different solid content and ash-sand ratio. Fu et al. [33,34] established the damage evolution model, damage constitutive model, and strength criterion of layered structure cemented paste backfill based on damage theory and absolute differential rule. The above damage constitutive model does not consider the influence of loading rate on the strength and damage evolution of the filling body.

Currently, the research on the mechanical properties of materials under loading rate mainly focuses on rock and concrete materials. However, there are few studies on the damage characteristics of filling materials with lower strength than rock and concrete and ductile failure characteristics, especially considering the loading rate based on AE parameters. As one of the mainstream dynamic non-destructive testing techniques, AE has attracted wide attention in studying material damage and failure characteristics. The uniaxial compressive strength test can effectively reflect the strength and failure characteristics of the filling body. AE technology can dynamically monitor the damage generation and development of the filling body during loading and provide data for experimental analysis [35,36,37]. When the filling body is subjected to load, the internal structure will deform or rupture, and accompanied by different sizes of energy, different frequencies of elastic wave release phenomenon is called AE of filling body [38]. To study the influence of loading rate on the characteristics of the aeolian sand paste filling body, this paper studies the influence of different loading rates (0.002 mm/s, 0.005 mm/s, 0.0075 mm/s, and 0.01 mm/s) on the strength, macroscopic failure characteristics, AE characteristic parameters and damage characteristics of aeolian sand paste filling body. The research results can provide experimental and theoretical references for the strength design of the filling body and the damage assessment under mining influence.

## 2. Experimental Materials and Methods

### 2.1. Experimental Materials

The selected experimental materials include aggregate (aeolian sand), cementitious materials (fly ash, cement), and water [39]. The aeolian sand is from Yuyang District in northern Shaanxi, the fly ash is from the filling station of Changxing Coal Mine in Yuyang District, the cement is ordinary Portland cement (OPC) 42.5, according to Chinese national standard GB175-2007, and the water is Xi’an ordinary tap water.

#### 2.1.1. Aggregate

**Figure 1:**
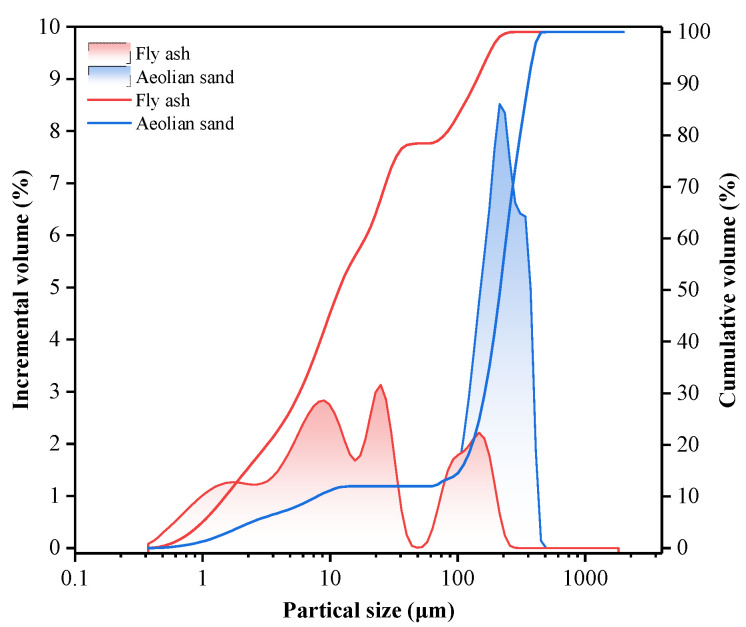
Particle size distribution of raw materials.

As the fourth monsoon product, aeolian sand mainly comprises lithic, feldspar, and quartz. The main chemical composition is shown in Table 1. As shown in Figure 1, the particle size distribution of aeolian sand is 0.412~493.6 μm, where d_10_ = 8.1 μm, d_50_ = 214.5 μm, d_90_ = 357.9 μm, and 100~400 μm accounts for about 80%. Aeolian sand’s uniformity coefficient (Cu) is about 30.3, and the optimum value of particle gradation is between 4 and 6, which conforms to the Talbot equation [40]. The particle size distribution curve shows that the coarse particle content is low, and the natural gradation is mostly discontinuous.

#### 2.1.2. Cementitious Materials

Cement is a cementitious material, and its main mineral components are C_2_S, C_3_S, C_3_A, C_4_AF, etc. The main chemical composition is shown in Table 2. The main hydration products are calcium hydroxide (CH), calcium silicate hydrate (C-S-H), calcium aluminate hydrate (C-A-H), and calcium alumino-ferrite hydrate (C-A-F-H).

As a kind of cementitious admixture, fly ash is used in mine filling, which can not only save cement and reduce filling costs but also improve the fluidity of pipeline slurry and the suspension performance of filling aggregate and effectively improve the late strength of the filling body. As shown in Figure 1, the particle size distribution of fly ash is 0.412~309 μm, of which d_10_ = 1.51 μm, d_50_ = 11.67 μm, d_90_ = 136.02 μm, 1~40 μm accounts for about 74%. The uniformity coefficient (C_u_) of fly ash is about 11.9, and the optimal particle gradation is between 4 and 6, which conforms to the Talbot equation [40]. The particle size distribution curve shows that the content of coarse particles is low, showing a discontinuous natural gradation. The main minerals are aluminosilicate, sponge-like vitreous, quartz, iron oxide, carbon particles, and sulfate. The main chemical composition is shown in Table 3.

### 2.2. Fabrication of Pecimens

The experimental ratio was fly ash: cement: aeolian sand = 35 wt.%: 12 wt.%: 53 wt.%, in which the solid mass concentration was 78% [41]. The mixing ratio of experimental materials can meet the engineering requirements by industrial field verification. The filling material was poured into the standard cylindrical mould with a diameter of 50 mm and a height of 100 mm after fully stirring according to the experimental ratio. A total of 12 filling specimens were made. After curing for 24 h, the mould was demoulded and put into the HWS constant temperature, and humidity curing box for 28 d, where the temperature was (20 ± 1) °C and the relative humidity was (95 ± 2)% [42].

### 2.3. Experimental Methods

**Figure 2:**
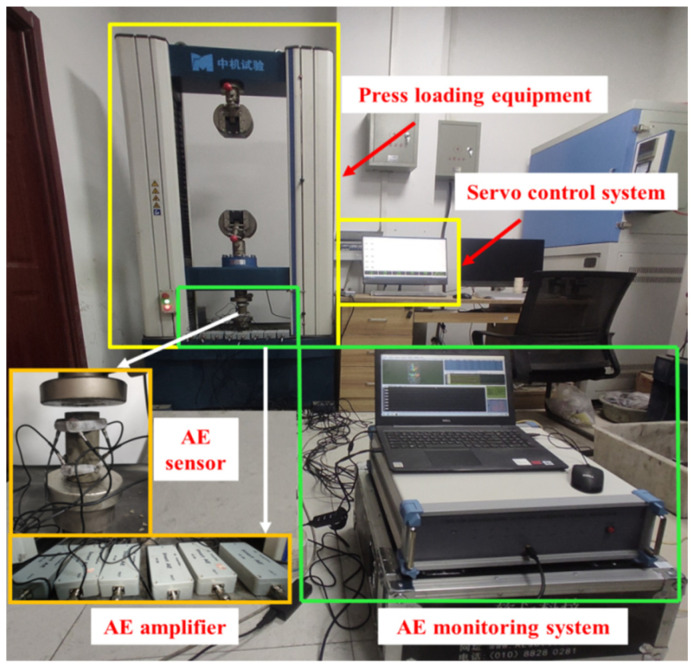
Experiments test system.

When the ASPM specimen reaches the specified curing time, the UCS test is carried out according to the standard for test methods of concrete physical and mechanical properties (GB/T 50081-2019) [43]. The experimental device is shown in Figure 2. The uniaxial compression tests of ASPM specimens with different loading rates were carried out by DNS100 electronic universal testing machine (SinoTest, Changchun, China). There is no uniform standard for the selection of loading rate in mechanical test experiments, and the stress recovery stage of rock mass in engineering sites after mining disturbance is mostly considered static loading, so it is feasible to use static loading research in the laboratory. Referring to the strain rate loading range of 10^−5^–10^−1^ mm/s in rock statics [44], different gradient loading rates were selected as 0.002 mm/s, 0.005 mm/s, 0.0075 mm/s, and 0.01 mm/s. The DS5-16B acoustic emission monitoring equipment of Beijing Softland Company was used to monitor the test process synchronously, and the parameters such as load, deformation, AE ringing count, and event number were recorded in real-time. Three AE sensors were arranged at 15 mm from the upper and lower ends of the specimen side, and vaseline was smeared on the contact part of the specimen and the probe to reduce the influence of AE caused by the friction between the probe and the specimen on the test. To avoid the loss of test data caused by the sensor shedding, 12 mm rubber reinforcement was used to fix the AE sensor on the specimen side. The AE threshold is 40 mV, and the sampling frequency is 3 MHz.

## 3. Results and Discussion

### 3.1. Mechanical Properties of the ASPM

#### 3.1.1. Results of UCS Analysis

The UCS test results of ASPM specimens under different loading rates are shown in Table 4. It shows that the standard deviation of the UCS of each group of specimens is slight, and the discreteness of the test results is small. When the loading rate increased from 0.002 mm/s to 0.005 mm/s, the average compressive strength of ASPM increased from 4.175 MPa to 4.641 MPa, with an increase of 11.17%. When the loading rate increased from 0.005 mm/s to 0.0075 mm/s, the average compressive strength of ASPM increased from 4.641 MPa to 5.191 MPa, with an increase of 11.85%. When the loading rate increased from 0.0075 mm/s to 0.01 mm/s, the average compressive strength of ASPM increased from 5.191 MPa to 5.408 MPa, with an increase of 4.18%. With the increase in loading rate, the change rate of peak strength of ASPM filling body increased first and then decreased, and the peak strength of ASPM filling body increased as a whole. It shows that the increase in loading rate has a noticeable strengthening effect on the peak strength of the ASPM filling body. This is due to the increase in loading rate, which shortens the failure time of the filling body and thus limits the full development of micro cracks, micropores, and other defects in the filling body. Therefore, the UCS increases macroscopically.

**Figure 3:**
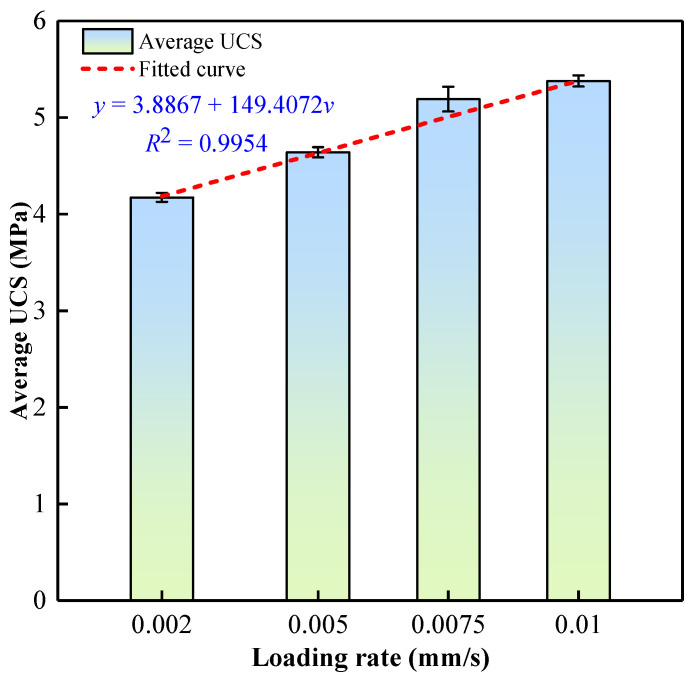
Relationship between average UCS and loading rate.

The experimental data were fitted, and it was found that the quantitative relationship between the average UCS of the specimen and the loading rate was more suitable for linear fitting, as shown in Figure 3. The fitting form is as follows:

(1) y=3.8867+149.4072v

**Figure 4:**
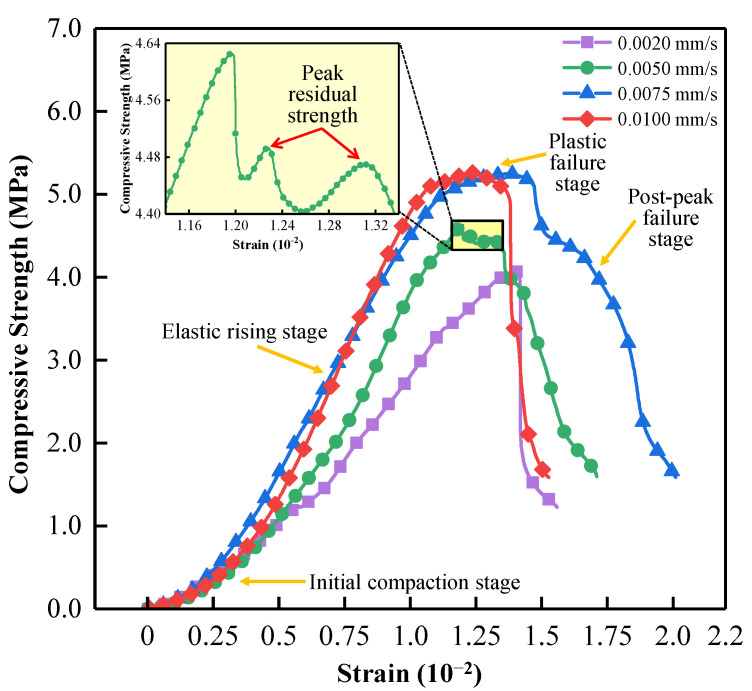
Stress–strain curve of ASPM specimen.

The typical stress–strain curves of the backfill specimens corresponding to different loading rates during uniaxial compression are shown in Figure 4. It can be seen from Figure that the ASPM specimen can be divided into four stages in the uniaxial compression process [36,45]: initial compaction stage (concave curve), elastic rise stage (oblique line), plastic failure stage (concave curve), and post-peak failure stage (post-peak curve). With the increased loading rate, the specimen experienced a shorter compaction stage and entered the linear elastic stage faster. Because the ASPM specimen is a kind of artificial production material, there are inevitably micropores and micro-cracks in the production process [46,47]. The higher the loading rate, the shorter the time internal defects are compacted, so it enters the linear elastic stage faster. The filling specimens have ductile failure characteristics and have residual strength after the peak [48,49]. In Figure, the stress–strain curve corresponding to 0.0050 mm/s appeared with two small peaks after the peak failure stage, indicating that ASPM specimens still have strong bearing capacity after reaching the peak failure [50].

#### 3.1.2. Results of Elastic Modulus Analysis

**Figure 5:**
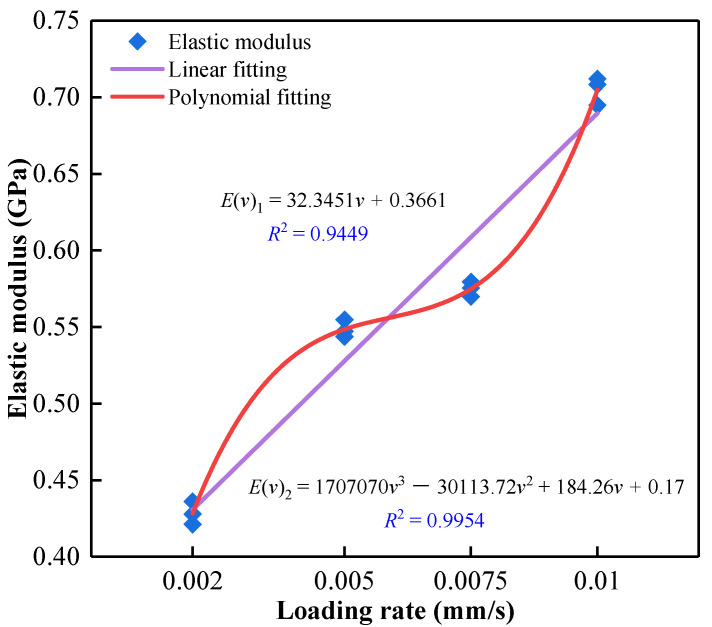
Relationship between elastic modulus and loading rate.

The elastic modulus of the filling body characterizes the deformation resistance of the material, and the physical essence is to characterize the binding force between the atoms of the material [51,52]. This paper defines the slope of the stress–strain curve’s elastic stage as the elastic modulus. The elastic modulus corresponding to different loading rates is shown in Table 4. Compared with the elastic modulus corresponding to the loading rate of 0.002 mm/s, the elastic modulus of the filling specimen increases by 29.65 % (0.005 mm/s), 35.44 % (0.0075 mm/s) and 65.53 % (0.01 mm/s) with the increase of loading rate, respectively. The elastic modulus of ASPM increases with the increase in loading rate, indicating that the increase in loading rate also has a strengthening effect on the stiffness of the ASPM filling body. Based on the above experimental results, a regression equation was established to characterize the quantitative relationship between elastic modulus and the loading rate of ASPM. Linear and polynomial methods fitted the relationship between the elastic modulus of ASPM and the loading rate. The fitting results are shown in Figure 5. It can be seen from Figure that the correlation coefficients *R*^2^ corresponding to linear fitting and polynomial fitting are 0.9449 and 0.9954, respectively, showing high fitting characteristics. However, the polynomial fitting degree is the highest, indicating that the polynomial fitting is more suitable to characterize the quantitative relationship between the loading rate and the elastic modulus of ASPM.

#### 3.1.3. Results of Failure Characteristics Analysis

**Figure 6:**
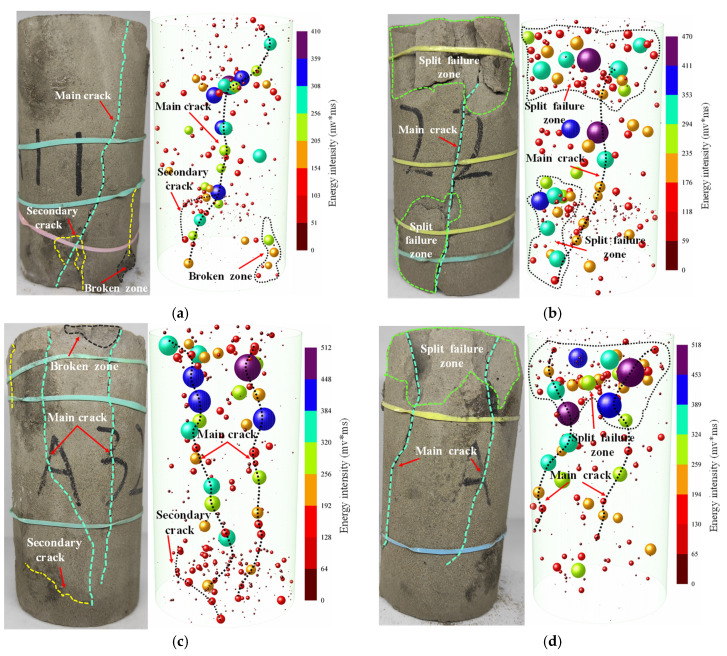
Failure mode and AE event points of ASPM specimens under different loading rates. (**a**) 0.002; (**b**) 0.005; (**c**) 0.0075; (**d**) 0.01.

Figure 6 shows the failure modes of ASPM specimens under different loading rates. When the loading rate is 0.002 mm/s and 0.005 mm/s, the specimen shows unidirectional shear failure, and the internal micro defects of the filling specimen have enough time to develop, making the internal defects develop fully. The cracks have sufficient time to penetrate each other, and the main cracks mostly accompany the secondary cracks. The specimen exhibits axial tensile failure when the loading rate increases to 0.0075 mm/s. When the loading rate is further increased to 0.01 mm/s, the internal micro pores and micro-cracks cannot be fully developed, and the weak surface in the specimen cannot be penetrated, so it can only develop along the respective dominant cracks. At the same time, under the action of positive pressure, the specimen has not only positive stress but also shear stress on the oblique section. At this time, the specimen will produce bidirectional shear failure. In summary, when the loading rate increases from 0.0020 mm/s to 0.01 mm/s, the failure mode of the ASPM specimen is mainly unidirectional shear failure → tensile failure → bidirectional shear failure. Therefore, the loading rate can have a particular impact on the failure mode of the ASPM filling body.

AE three-dimensional positioning can effectively reflect the formation location and spatial evolution of micro cracks in filling specimens and evaluate the damage and failure of the specimen [53,54]. The ball in the diagram represents the event point, and the ball size represents the energy size. AE has an apparent response to the loading rate. At a low loading rate, the AE events inside the specimen are more dispersed, and the significant energy event points are less. This is because the specimen has more time to make each part uniformly compressed at low-speed loading, so the AE energy is primarily tiny. Under the high loading rate, the AE events in the specimen are more concentrated, and the large energy points are more. This is because the high loading rate makes the rapid internal response of the filling body, and the AE events begin to occur at the weak surface. The AE events are more concentrated due to the independent development of the weak surface. The energy generated by the increase of the loading rate is also increased accordingly. It can be seen from the graph that under the high loading rate, the large energy events are primarily concentrated in the upper part, and the lower part is more distributed with small energy events. It shows that the internal energy accumulates to a certain extent during the loading process and releases energy first in the upper part to produce cracks. The cracks propagate from top to bottom, and the internal energy releases slowly with the crack propagation. It is found that the AE event points are consistent with the experimental crack development.

### 3.2. Results of AE Analysis

As the number of oscillations exceeds the AE threshold, the AE ring count can further characterize filling specimens’ internal micro-fracture and damage evolution. The AE cumulative ringing count refers to the cumulative value of the ringing count of AE in an AE process. This parameter can describe the total strength of AE and the accumulation of internal damage to materials, which is the external manifestation of the accumulation effect of internal structural changes in materials. Through AE monitoring of ASPM specimens under uniaxial compression test at different loading rates, the internal defects and damage can be reflected, and the failure of the filling body can be predicted [55].

#### AE Ringing Count Analysis

**Figure 7:**
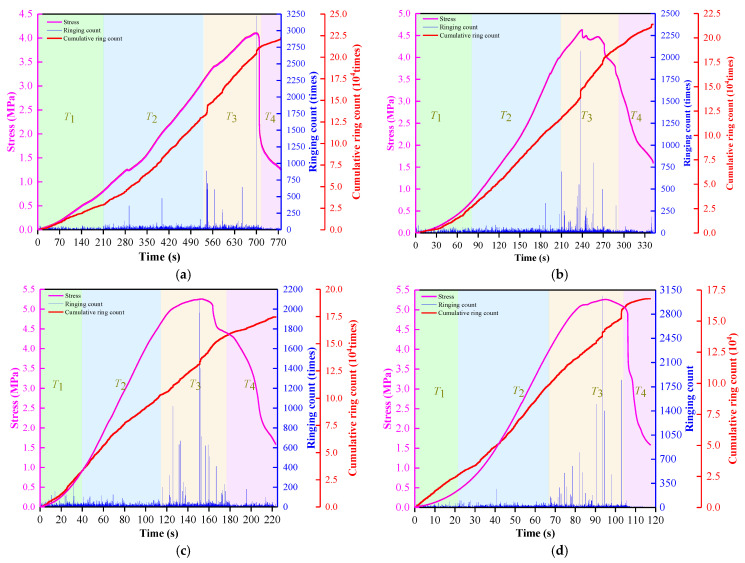
AE ringing counts, stress and time curves of ASPM specimens. (**a**) 0.002; (**b**) 0.005; (**c**) 0.0075; (**d**) 0.01.

The AE ringing counts, stress, and time relationships of the filling specimens under uniaxial compression at different loading rates are shown in Figure 7. It can be seen from Figure 7 that the number of AE ringing reaches the maximum when the peak stress approaches. The reason is that the micro-cracks initiate, develop and expand after the micro-pore compaction in the early stage of the specimen. The macroscopic penetrating cracks are formed near the peak stress. The accumulated energy is released rapidly so that the AE ringing count signal value is suddenly increased to the peak value, which can be used as a precursor signal to determine the failure of the filling specimen [50].

According to the change rule of AE ringing count, the whole loading process can be divided into four stages: initial active stage, pre-peak rise stage, active stage, and post-peak stability stage.

**Figure 8:**
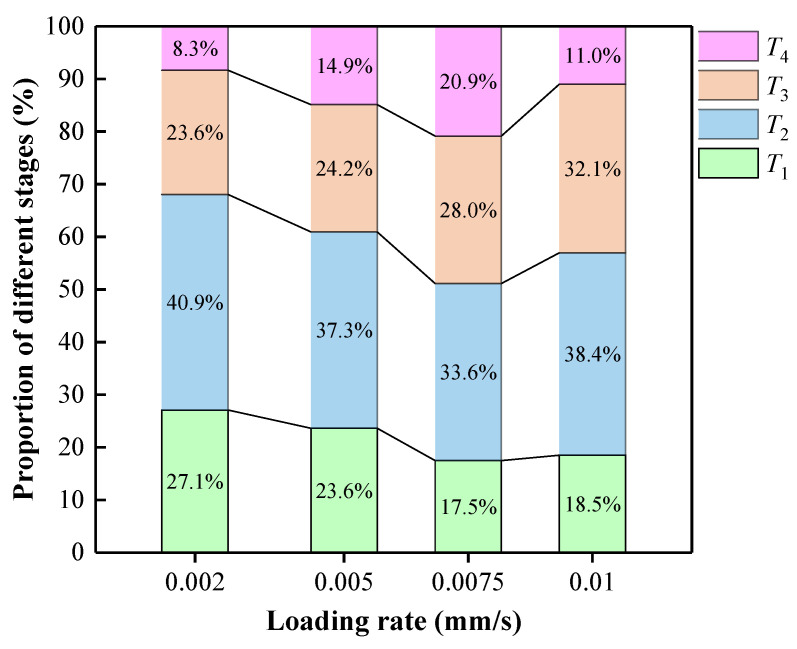
The proportion of each stage.

(1) The initial active stage *T*_1_ (A11: 0~211 s, A21: 0~81 s, A32: 0~39 s, A43: 0~22 s): This stage corresponds to the initial compaction stage of the stress–strain curve. The internal pore defects of the specimen are compacted, accompanied by friction between the filling materials, resulting in a sporadic sharp increase in the ringing count, but the sudden increase is small. This stage accounts for about 27.1%, 23.6%, 17.5%, and 18.5% of the process, as shown in Figure 8. The proportion of the initial active stage was negatively correlated with the loading rate. The results show that the increase in loading rate makes the filling specimen enter the next stage faster, which is consistent with the findings in 3.1.1.
(2) The pre-peak rise stage *T*_2_ (A11: 211~530 s, A21: 81~209 s, A32: 39~114 s, A43: 22~67 s): This stage corresponds to the elastic stage of the stress–strain curve. With the increase in loading, new cracks begin to initiate and expand and continuously release AE signals. There is no mutation in the ringing count of AE at this stage, and the overall ringing count is stable and high, so the cumulative ringing count curve can be seen that the whole is in a steady growth stage. This stage accounts for about 40.9%, 37.3%, 33.6%, and 38.4% of the process, as shown in Figure 8.
(3) The active stage *T*_3_ (A11: 530~714 s, A21: 209~292 s, A32: 114~177 s, A43: 67~104 s): After the previous energy savings to the stage of AE parameters into the active phase. In the pre-peak stage, the ringing number increased sharply with the increase of axial stress, reaching the maximum near the peak, and then decreased sharply with the decrease of axial stress after the peak. This stage accounts for about 23.6%, 24.2%, 28.0%, and 32.1% of the process, as shown in Figure 8. The proportion of the active stage is positively correlated with the loading rate, indicating that the increase in the loading rate that makes the internal energy in the compression process cannot be released until close to the peak. The higher the loading rate is, the more energy is released, so it can be seen that the proportion of this stage is gradually increasing.
(4) The post-peak stability stage *T*_4_ (A11: 714~779 s, A21: 292~342 s, A32: 177~223 s, A43: 104~117 s): This stage corresponds to the post-peak failure stage of the stress–strain curve. After the sudden increase and decrease of the ringing count in the previous stage, the ringing count in this stage is at a low level as a whole. Because there is some friction and slipping between the fracture surfaces after the failure of the specimen, there is a small range of growth at some time, but it does not affect the overall development trend. This stage accounts for 8.3%, 14.9%, 20.9%, and 11.0% of the process, as shown in Figure 8.

**Figure 9:**
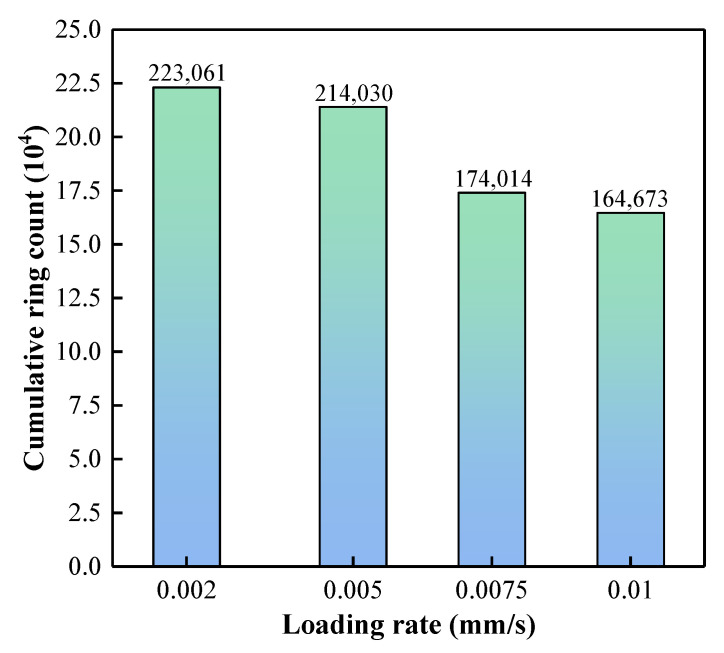
Relationship between cumulative ringing count and loading rate.

As shown in Figure 9, it can be seen that with the increase in loading rate, the AE cumulative ringing count negatively correlates with the loading rate. It is concluded that when the loading rate is less than 0.002 mm/s, the total number of AE cumulative ringing will be at a high level. When the loading rate exceeds 0.01 mm/s, the total number of AE cumulative ringing will be further reduced.

## 4. Establishment of Damage Constitutive Model of the ASPM

### 4.1. Fitting of AE Cumulative Ringing Count and Strain

The analysis of the above experimental results shows that the AE characteristics are closely related to the development of microcracks in ASPM specimens. Cracks and defects inside the filling specimen are essential factors affecting its mechanical properties. Therefore, there is an inevitable connection between the AE cumulative ringing count and the mechanical properties of the filling specimen.

According to the experimental data, the relationship between strain and time is fitted. That is the following relationship between *ε* and time:

(2) $$\varepsilon =kt+\varepsilon_{0}$$

The formula: *ε* is the strain of the filling body specimen; *k* is the strain rate; *t* is time; *ε*_0_ is the initial strain of the filling body, obtained by linear fitting experimental data.

The Boltzmann function can express the relationship between the measured cumulative ringing count and time [56,57], that is:

(3) $$N=\frac{A-B}{1+\exp\left(\frac{t-C}{G}\right)}+B$$

The formula: *N* is the AE cumulative ringing count in the loading stage; *t* is time; *A*, *B*, *C*, and *G* are all fitting parameters.

According to the experimental results, the total number of AE cumulative ringing decreases with the increase of loading rate, the loading rate *v* is introduced, and the fitting relationship is further modified as follows:

(4) $$N=\frac{A-B}{1+\exp\left[\frac{v\left(t-C\right)}{G}\right]}+B$$

The formula: *v* is the uniaxial loading rate of the filling specimen.

**Figure 10:**
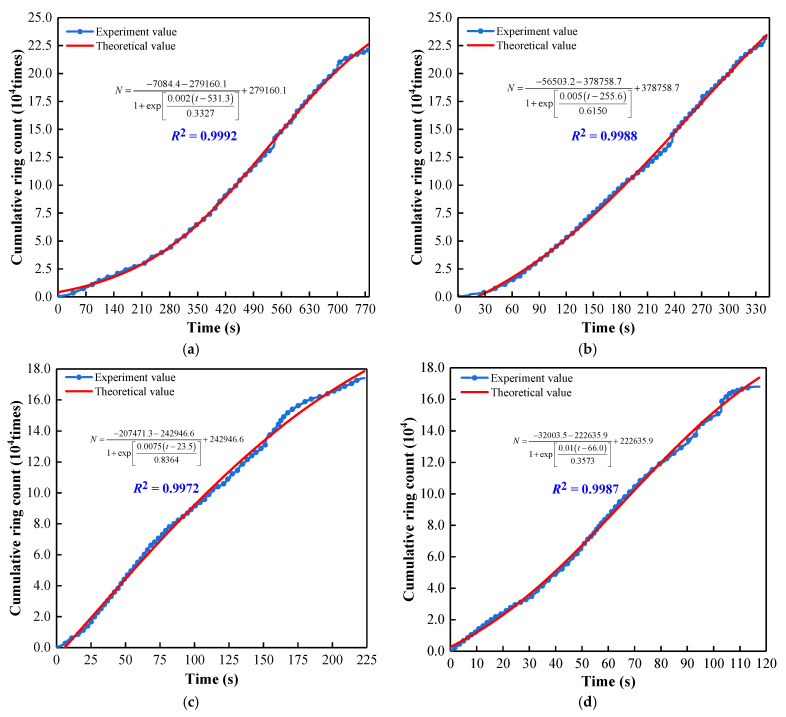
Fitting of the AE cumulative ringing count with time. (**a**) 0.002; (**b**) 0.005; (**c**) 0.0075; (**d**) 0.01.

Using the Formula (4) to fit, as shown in Figure 10, the correlation coefficients corresponding to different loading rates are 0.9992 (0.002 mm/s), 0.9988 (0.005 mm/s), 0.9972 (0.0075 mm/s) and 0.9987 (0.01 mm/s). Therefore, the function can represent AE cumulative ringing count and time variation.

Formulas (2) and (4) can be obtained:

(5) $$N=\frac{A-B}{1+\exp\left[\frac{v\left(\varepsilon -\varepsilon_{0}-kC\right)}{kG}\right]}+B$$

(6) $$\varepsilon =k\left[\frac{G}{v}\ln\left(\frac{A-N}{N-B}+C\right)\right]+\varepsilon_{0}$$

Formulas (5) and (6) establish the coupling relationship between cumulative ringing counts and strain of ASPM specimens under different loading rates.

### 4.2. Establishment of the ASPM Damage Model

In this paper, AE ringing count and AE cumulative ringing count are selected as characteristic parameters to characterize the damage characteristics of filling specimens during compression.

Kachanov [58] proposed the concept of damage variable *D* and defined it as:

(7) $$D=\frac{A\prime }{A}$$

The formula *A* is the total area of micro defects on the bearing section and *A’* is the fracture area when there is no initial damage.

Considering that it is difficult to determine the effective bearing area of damaged materials, Lemaitre [59] proposed the strain equivalence hypothesis, that is, to indirectly measure the damage through effective stress:

(8) σ=σ*(1−D)=Eε(1−D)

Formula: *σ* is nominal stress, σ* is effective stress, *E* is the elastic modulus of the filling body, and *ε* is strain.

Assuming that the AE cumulative ringing count is *N_f_* when the whole section A of the non-destructive material is completely damaged, the AE ringing count *N_w_* when the unit area is damaged is:

(9) $$N_{w}=\frac{N_{f}}{A}$$

When the cross-section damage reaches *A’*, the AE cumulative ringing count is:

(10) $$N_{d}=N_{w}A\prime =\frac{N_{f}}{A}A\prime$$

Formulas (7) and (10) show that the relationship between the damage variable and AE cumulative ringing count is:

(11) $$D=\frac{A\prime }{A}=\frac{N_{d}}{N_{f}}$$

During the experiment, due to the insufficient stiffness of the testing machine or the different failure conditions set, the testing machine was stopped when the specimen was not completely damaged (the damage variable *D* does not reach 1). There is still a specific residual strength of the specimen. Therefore, the modified damage variable is:

(12) $$D=D_{u}\frac{N_{d}}{N_{f}}$$

Formula: *D_u_* is the critical value of the damage.

For the convenience of calculation, the critical damage value *D_u_* is:

(13) $$D_{u}=1-\frac{\sigma_{c}}{\sigma_{pk}}$$

Formula: *σ_pk_* is peak strength, *σ_c_* is residual strength.

The coupling relationship between cumulative ringing count *N*, damage variable *D*, and stress *σ* of ASPM at different loading rates can be obtained by combining Formulas (5), (8), (12), and (13) as follows:

(14) $$D=(1-\frac{\sigma_{c}}{\sigma_{pk}})\frac{N_{d}}{N_{f}}$$

(15) $$\sigma =E\varepsilon \left(1-D\right)=E\varepsilon \left[1-(1-\frac{\sigma_{c}}{\sigma_{pk}})\frac{N_{d}}{N_{f}}\right]$$

Formula: *N_f_* is the cumulative ringing count produced at the end of the experiment,$N_{d}=\frac{A-B}{1+\exp\left[\frac{v\left(\varepsilon -\varepsilon_{0}-kC\right)}{kG}\right]}+B$.

### 4.3. Model Validation and Discussion

To verify the rationality and effectiveness of the model, combined with experimental data, the strain-time curve and the cumulative count-time curve of AE ringing are fitted, respectively. The statistical fitting parameters are shown in Table 5. They are substituting the fitting parameters into formulas (6), (4) and (15), the comparison between AE cumulative ringing count and strain, damage variable and strain, stress–strain fitting based on AE ringing count, and measured stress–strain under ASPM uniaxial compression can be determined.

**Figure 11:**
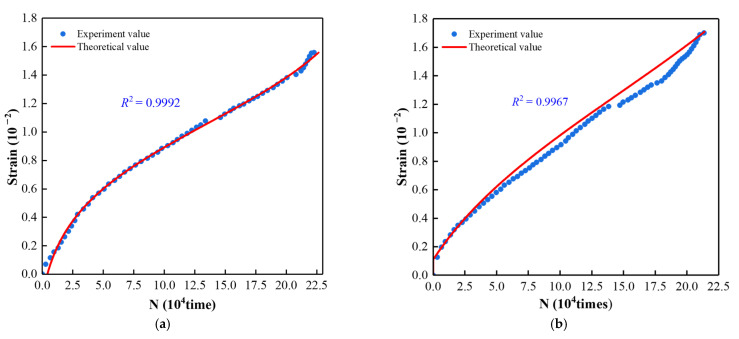

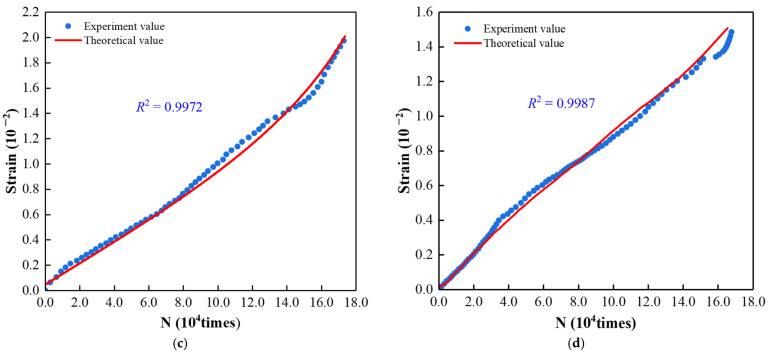
Comparison of Experiment value and Theoretical value of *N* and *ε.* (**a**) 0.002; (**b**) 0.005; (**c**) 0.0075; (**d**) 0.01.

As shown in Figure 11, the comparison between the experimental and theoretical results shows that the theoretical and experimental curves of strain and AE cumulative ringing count have a high matching, which indicates that the coupling relationship between AE cumulative ringing count and strain considering loading rate is appropriate. The model can provide a reference for the deformation prediction of the same type of filling materials.

**Figure 12:**
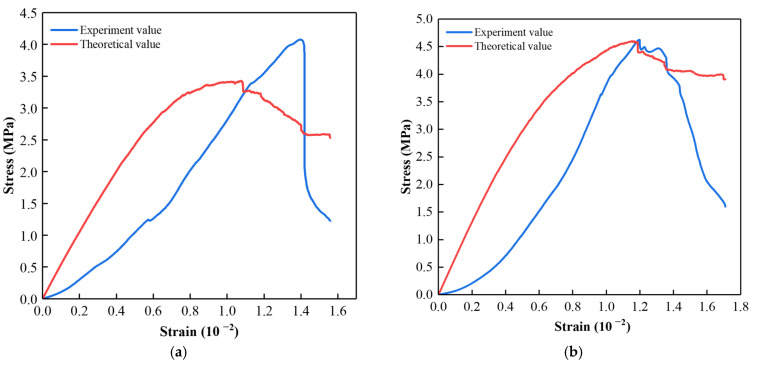

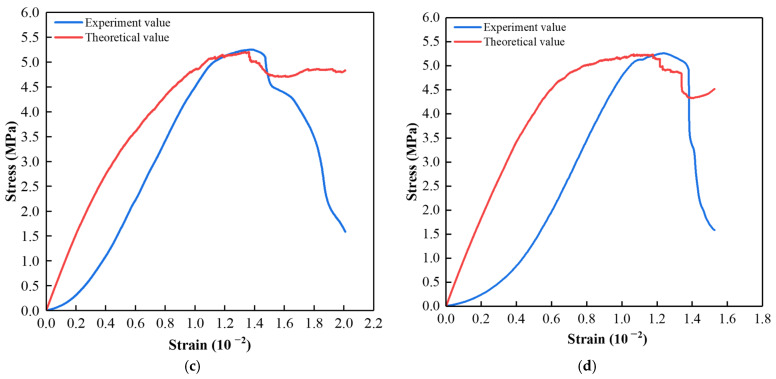
Comparison of Experiment value and Theoretical value of *σ* and *ε.* (**a**) 0.002; (**b**) 0.005; (**c**) 0.0075; (**d**) 0.01.

Figure 12 compares the stress–strain curves of ASPM specimens under different loading rates and the experimental results. At a low loading rate, the theoretical stress peak is smaller than the measured stress peak, and the peak strain is ahead of the measured value. With the increased loading rate, the theoretical peak stress and peak strain are close to the experimental results, which can better reflect the failure process of ASPM specimens from linear elastic transition to plastic deformation. Compared with the experimental results, the established model cannot effectively reflect the compaction stage of ASPM specimens under different loading rates, and the theoretical residual strength after the peak is greater than the experimental value.

**Figure 13:**
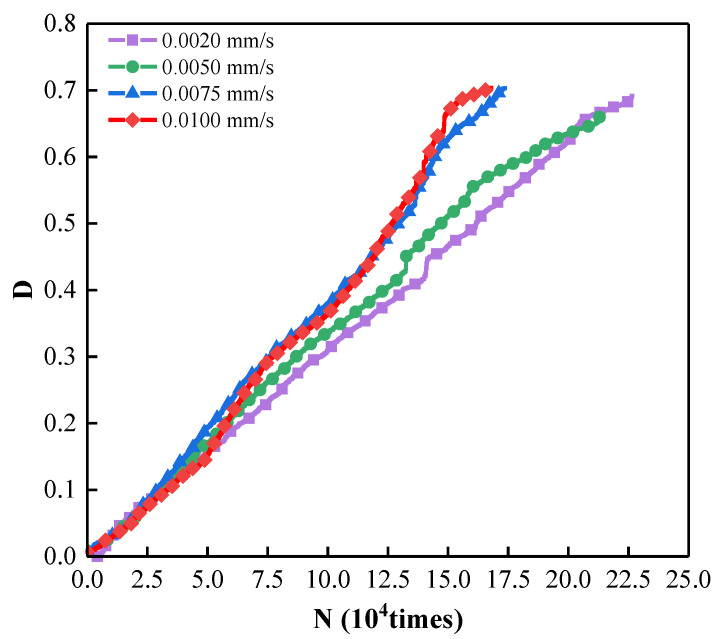
Relationship between *N* and *D*.

Figure 13 shows the relationship between AE cumulative ringing counts and damage variables at different loading rates. It can be seen that the higher the loading rate is, the smaller the final AE cumulative ringing counts are. The final damage values at different loading rates are about 0.7, which again shows that ASPM specimens still have a specific residual strength at the end of the experiment. In the Figure, the higher the loading rate is, the greater the slope of the curve is, indicating that the higher the loading rate per unit time is, the greater the damage to the ASPM specimen is.

Overall, the strain, stress, and damage variables of ASPM specimens agree with the measured and simulated cumulative counts of AE ringing, which proves the rationality and effectiveness of the coupling model.

## 5. Conclusions

(1) Based on the AE monitoring technology, this study analysed the comprehensive influence of loading rate on the strength, deformation, AE characteristics and damage characteristics of ASPM, a lower strength filling material. The following conclusions can be drawn: The loading rate strengthens the uniaxial compressive strength and elastic modulus of ASPM specimens. When the loading rate increased from 0.002 mm/s to 0.01 mm/s, the uniaxial compressive strength increased by 29.5% and the elastic modulus increased by 65.53%. The average uniaxial compressive strength and loading rate are linear function distributions, and the elastic modulus and loading rate are polynomial. The failure mode is mainly unidirectional shear failure when there is a low loading rate. When the loading rate is high, it presents tensile failure mode, and the loading rate further increases, showing a bidirectional shear failure mode.
(2) Under a low loading rate, the internal event points of ASPM specimens are more dispersed, and the large energy points are less. Under a high loading rate, the internal event points are more concentrated, and the large energy points are more. The large energy events are primarily concentrated in the upper part, and the lower part is more distributed with small energy events. AE events correspond to experimental crack development and failure.
(3) According to the change in AE ringing count, the loading process is roughly divided into four stages: initial active, pre-peak rise, active, and post-peak stability. The loading rate increased from 0.002 mm/s to 0.01 mm/s, the proportion of the initial active stage decreased from 27.1% to 18.5%, and the active stage increased from 23.6% to 32.1%. The total number of AE cumulative ringing decreases with the increase in loading rate.
(4) By fitting the strain of ASPM specimen, AE cumulative ringing count, and time, the relationship model between AE cumulative ringing count and strain of ASPM specimen considering loading rate is constructed; the damage and stress coupling model of ASPM specimens under different loading rates based on AE cumulative ring count can better reflect the damage evolution process of ASPM under higher loading rates. The construction of the coupling model under a low loading rate remains to be further studied.

## Figures and Tables

**Table 1:** Main chemical composition of Aeolian sand.

Composition	CaO	Al_2_O_3_	SiO_2_	Fe_2_O_3_	K_2_O	Others
Content, %	5.3	10.3	67.8	5.8	7.5	3.3

**Table 2:** Main chemical composition of Cement.

Composition	CaO	Al_2_O_3_	SiO_2_	Fe_2_O_3_	MgO	Others
Content, %	65.08	5.53	22.36	3.46	1.27	2.30

**Table 3:** Main chemical composition of Fly ash.

Composition	CaO	Al_2_O_3_	SiO_2_	Fe_2_O_3_	K_2_O	Others
Content, %	10.51	17.82	55.46	5.32	2.81	8.08

**Table 4:** UCS of the ASPM specimens with different loading rates.

Specimen Number	Loading Rate mm/s	Compressive Strength/MPa	Average Compressive Strength/MPa	Standard Deviation	Elastic Modulus/GPa
A11	0.0020	4.151	4.175	0.0461	0.4279
A12		4.239			0.4213
A13		4.134			0.4361
A21	0.0050	4.712	4.641	0.0531	0.5548
A22		4.628			0.5437
A23		4.584			0.5472
A31	0.0075	5.034	5.191	0.1282	0.5698
A32		5.348			0.5796
A33		5.192			0.5755
A41	0.0100	5.377	5.408	0.0580	0.6948
A42		5.489			0.7120
A43		5.357			0.7083

**Table 5:** Fitting parameters of different loading rates.

Specimen Number	Loading Rate mm/s	Fitting Parameters							*σ_pk_*	*σ_c_*
		A	B	C	G	*R* ^2^	*k*	*ε_0_*		
A11	0.0020	−7084.4	279,160.1	531.3	0.3327	0.9992	2.0 × 10^−5^	1.3 × 10^−6^	4.151	1.225
A12		−34,910.9	262,770.0	367.7	0.3274	0.9988	2.0 × 10^−5^	9.6 × 10^−7^	4.239	1.251
A13		−47,599.4	397,075.6	720.4	0.6441	0.9975	2.0 × 10^−5^	7.8 × 10^−7^	4.134	1.285
A21	0.0050	−56,503.2	378,758.7	255.6	0.6150	0.9988	5.0 × 10^−5^	4.5 × 10^−6^	4.712	1.597
A22		−29,520.9	219,770.7	146.0	0.3434	0.9989	5.0 × 10^−5^	5.3 × 10^−6^	4.628	1.364
A23		−45,896.4	268,022.9	163.1	0.4310	0.9994	5.0 × 10^−5^	5.6 × 10^−6^	4.584	1.362
A31	0.0075	−34,700.4	198,133.6	79.8	0.3045	0.9981	7.5 × 10^−5^	7.5 × 10^−5^	5.034	1.489
A32		−207,471.3	242,946.6	23.5	0.8364	0.9972	7.5 × 10^−5^	1.1 × 10^−5^	5.348	1.587
A33		−110,341.6	256,808.5	88.1	0.6429	0.9975	7.5 × 10^−5^	8.4 × 10^−6^	5.192	1.533
A41	0.0100	−41,430.5	185,097.7	49.8	0.2979	0.9995	1.0 × 10^−4^	1.2 × 10^−5^	5.377	1.609
A42		−16,918.1	236,993.5	96.2	0.3439	0.9983	1.0 × 10^−4^	1.2 × 10^−5^	5.489	1.598
A43		−32,003.5	222,635.9	66.0	0.3573	0.9987	1.0 × 10^−4^	1.5 × 10^−5^	5.357	1.587

## Data Availability

The data used to support the findings of this study are included in the article.
